# Supplementary material for: Preparation of High Water-Soluble Trichoderma Co-Culture Metabolite Powder and Its Effects on Seedling Emergence Rate and Growth of Crops
Source: J Fungi (Basel). 2023 Jul 20;9(7):767. doi: 10.3390/jof9070767 (PMC10381636; doi:10.3390/jof9070767)
Supplement: Supplementary file 1 [file jof-09-00767-s001.zip › jof-2506940-SI.pdf]

Journal of Fungi

MDPI

Supplementary materials 1

1. Fungal Strains

Strain	Strain number	Collection location
T. cf.asperelloides	CTCCSJ-A-CM100Z4-1	Shanghai Chongming Wenzhou Mandarin bark
T. cf. afroharzianum	CTCCSJ-W-RW10569-1	Hainan - Wetland - soil
T. cf. kunmingense	CTCCSJ-A-GDFS1009-1	Guangdong - farmland - soil
T. cf. asperelloides	CTCCSJ-W-SBW10264-1	Hainan - Wetland - soil

We tested the strains for salt tolerance, high-temperature tolerance and chitinase activity. High-temperature-tolerant Trichoderma Z4-1, high-salt-tolerant Trichoderma 1009-1, high-chitinase-producing Trichoderma 10569-1, and the mixed fermentation of Trichoderma echinospora 10264-1 and the abovementioned Z4-1, 1009-1 and 10569-1 were the best for seed germination. The above four strains were selected for subsequent experiments.The accession number of 1009-1 is OP799503;The accession number of 10264-1 is OP799504;The accession number of 10569-1 is OP799505;The accession number of Z4-1 is OP799506.

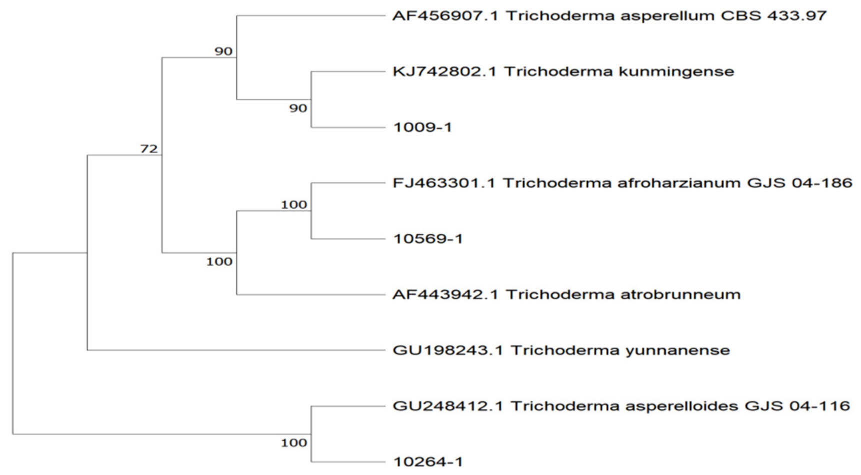

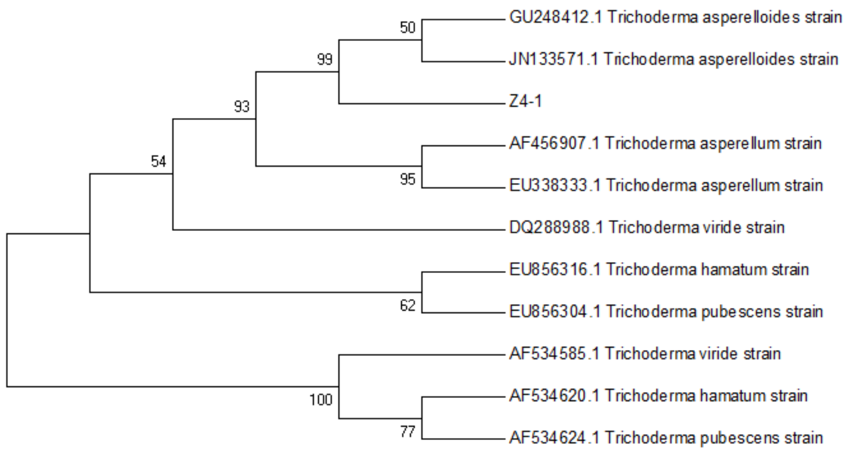

Z4-1 was identified as *Trichoderma* asperelloide.1009-1was identified as *Trichoderma* . cf. Kunmingense.10569-1 was identified as *Trichoderma*. cf. Afroharzianum.10264-1 was identified as *Trichoderma* . cf. asperelloides

Supplementary materials 2

2. Basic characteristics of the ten carriers

Pregelatinized starch: Sodium carboxymethyl starch (C6H10O5)n (n=300~1000) containing 5% free amylose, 15% free amylopectin and 80% unmodified starch; pH=4.5-7.0.

β-Cyclodextrin: Cyclomaltose and cycloheptadrin composed of 7 glucose residues in the β-A ring formed by the binding of 1,4-glycosidic bonds; $C42H70O35.xH2O$, pH=5.0-8.0.

XW-maltodextrin: Starch, polysaccharides, tetrasaccharide or oligosaccharides mixed with the tetrasaccharides maltose and glucose $(C6H10O5)n$, pH=4.5-6.5.

LG-maltodextrin: Starch, polysaccharide, tetrasaccharide or oligosaccharide above tetrasaccharide, maltose and mineral mixtures such as glucose, calcium and iron; $(C6H10O5)n$, pH=4.5-6.5.

Dextrin: Starch, polysaccharide, tetrasaccharide or oligosaccharide above tetrasaccharide, and small amounts of maltose and glucose, calcium and iron mixture; $(C6H10O5)n$.

Jiayi powder: A high-tech composite made of a variety of minerals, inorganic salts, modified starch and other auxiliary materials through special processing.

Jiawei powder: Minerals and soluble starch; pH=5.5-8.0.

Disclaimer/Publisher's Note: The statements, opinions and data contained in all publications are solely those of the individual author(s) and contributor(s) and not of MDPI and/or the editor(s). MDPI and/or the editor(s) disclaim responsibility for any injury to people or property resulting from any ideas, methods, instructions or products referred to in the content.
